# Bibliographical Notices

**Published:** 1844-03-09

**Authors:** 


### BIBLIOGRAPHICAL NOTICES.

*Pharmacologia, being an Extended Inquiry into the Operations of Medicinal Bodies, upon which are Founded the Theory and Art of Prescribing.* By J. A. Paris, M. D., &c. *From the Ninth London Edition. With Notes.* By Charles A. Lee, M. D., A. M., Late Professor (Elect) of Materia Medica and Medical Jurisprudence, in the University of the City of New York; Consulting Physician to the Northern Dispensary of New York; Member of the New York Lyceum of Natural History, &c. &c. &c. Octavo. pp. 353. New York: Harper & Brothers. 1844.

The Pharmacologia of Dr. Paris has long been a standard work. It is thirty-two years since it was first published, and it has now reached the ninth edition. In that long period so numerous and important have been the discoveries and improvements in Physiology, Materia Medica, and Chemistry, that the author has found it necessary to rewrite the work in order to accommodate it to the existing state of science. Otherwise it is essentially the same as in former editions, with the exception of the "Second Part," which comprised the Materia Medica, which is now omitted. This alteration, (we cannot call it improvement) lessens the size and cost of the book, and in the estimation of most persons, probably, the value too; for this, however, neither the editor nor publishers are responsible. The editor, Dr. Lee, is favourably known in American Medical Literature. The *Notes* which he has appended, without materially increasing the size of the volume, contain some curious and instructive matter, aptly expressed, and consequently enhance its value.

*Anatomical Atlas, Illustrative of the Structure of the Human Body.* By Henry H. Smith, M. D., &c. *Part Second. Containing Ninety-one Figures.*

The present number treats of the "dermoid and muscular sytems," and fully sustains the favourable opinion of the work expressed in a former number of the Examiner, on the appearance of the first part. It is creditable to the author and to the artists who have been employed on it.

---

*Report of the Pennsylvania Hospital for the Insane, for the year* 1843. By Thomas S. Kirkbride, M. D., Physician to the Institution.

This excellent Institution seems likely to realise the most sanguine hopes entertained by its friends and patrons. The "managers" are gentlemen of intelligence and probity, and the physician, to whose care the patients are consigned, we know to be all that such an one ought to be.

The following extracts exhibit the condition of the Institution, past and present, and the class of patients deemed proper for admission.

Since the date of the last report, we have had the gratification to witness no less than sixty-eight of our fellow beings, who had been afflicted with mental disease,—some from the most distant sections of the Union,—return to their families and to society in the full enjoyment of health; many others with health materially improved, and new means of enjoyment opened to those whose maladies have proved intractable.

During the same period, however, we have had to regret the admission of an unusual number, labouring under severe physical disease, and a few in a state of prostration, that prevented our doing more than endeavouring to soothe their last moments.

At the date of the last report, there were one hundred and eighteen patients in the Hospital, since which one hundred and forty have been admitted, and one hundred and twenty-six have been discharged or died, leaving one hundred and thirty-two under care at the close of the year.

Since the opening of this Hospital, three years since, there has been a steady increase in the number of patients admitted, and the number under care at one time has been constantly augmenting, as will be seen from the following tables, which embrace all the cases that had not been in the Hospital in the city:

| | |
|---|---|
| 1. In 1841, there were admitted . | 83 patients. |
| 1842, " " | 111 " |
| 1843, " " | 140 " |
| 2. The average number in 1841, was . | 104 |
| " " 1842, " . | 114 |
| " " 1843, " . | 132 |
| 3. The highest number at one time, in 1841, was . | 116 |
| " " " 1842, " . | 127 |
| " " " 1843, " . | 145 |
| 4. The total number under care, in 1841, was . | 176 |
| " " " 1842, " . | 238 |
| " " " 1843, " . | 258 |

Of those discharged during the year 1843, were—

| | |
|---|---|
| Cured . . . . . | 68 |
| Much Improved . . . . . | 7 |
| Improved . . . . . | 14 |
| Stationary . . . . | 20 |
| Died . . . . . | 17 |
| Total, | 126 |

Of the patients discharged "Cured," thirty-four were residents of the Hospital, not exceeding three months; twenty-three, between three and six months; eight, between six months and one year; and three, for a longer period than one year.

Of those discharged "Much Improved," three were under treatment not exceeding three months; two, between three and six months; and two between six months and one year.

Of the "Improved," five were under care less than three months; three, between three and six months; three, between six months and one year; and three for a longer period than one year.

Of those discharged "Stationary," five were in the house less than three months; five, between three and six months; three, between six months and one year; and seven over one year.

Among those discharged without being well, were several patients whose removal was premature, and in whose cases, a proper trial of treatment would probably have resulted in recoveries. Of these, four are registered "Much Improved," three "Improved," and four "Stationary." A few we have learned continued to convalesce, and ultimately became quite well. They have been reported as they were when they left the Hospital.

Ten males and seven females have died during the year. Of these, three were cases of apoplexy (two being third attacks), three were cases of dysentery, of a violent form, with which several of our debilitated patients were attacked during the past summer; two died from abscess of the kidneys; one from chronic bronchitis; one, cancer of the intestines; one, ulceration of the intestines of long standing; one, erysipelas, with effusion in the thorax; one, gradual wasting of the powers of life; one, sudden death, in a case of enlargement of the heart, with ossification of the aorta; two were cases of inflammation of the brain, dying on the fourth day after admission; and one patient who had been under high excitement for some time, fell into a state of collapse, and died a few hours after reaching the Hospital.

From the above abstract it will be observed, that more than one-half of the deaths have resulted from chronic diseases of a dangerous character, most of which existed before the patients entered the Hospital and between which and their insanity there was, in several instances, a direct connexion. This was particularly so with the cases of apoplexy and abscess of the kidneys. The case of cancer of the intestines was one of those latent forms of disease, occasionally observed among the insane, going on steadily to near their fatal termination, often without a complaint from the patient, aud with hardly a symptom likely to lead to a correct diagnosis.

The cases of inflammation of the brain, it is scarcely necessary to say, should never have been brought to the Hospital. This disease, under the most favourable circumstances of rest and quiet, is of a highly dangerous character; but when a patient is subjected to the exposure and excitement, attendant upon a rough journey, the result can hardly prove different from that which occurred in the cases which reached us.

It is very desirable that the insane should always be promptly placed under treatment—the favourable termination of the disease and its duration being often dependant upon such a course; but it is of the utmost importance that a patient should never be sent from home till the character of his illness is positively made out, nor till there is an absolute certainty that the individual is not labouring under the delirium of phrenitis, or some low form of fever.

With the exception of several cases of dysentery last summer, already referred to, our patients have had a remarkable exemption from all acute disease, since the opening of the Hospital.

All classes of insane persons, without regard to the duration of the disease or of its curability, are admitted into this Institution. Idiots, however, are not received; and for the epileptic, a special agreement should be made.

Cases of Mania à Potû, are not received into this Hospital—but into that in the city, *exclusively.*

---

*Lehrbuch der Allgemeinen Anatomie des Menschen.* Nach eigenen Untersuchungen zum Gebrauche bei Vorlesungen, sowie zum Selbststudium fuer praktische Aerzte und Wundaerzte: bearbeitet von Victor Bruns, Doctor der Medicin und Chirurgie, Professor an dem Collegium Anatomico-Chirurgicum zu Braunschweig, praktischem Arzte und Wundaerzte. 8vo. S. 498. Braunschweig: 1841.

*Manual of the General Anatomy of Man, &c. &c.* By Victor Bruns, &c. &c. 8vo. pp. 398. Brunswick: 1841.

The recent histological researches with the microscope and the valuable information emanating from them have led the author to the publication of this work. Dr. Bruns is himself an experienced microscopist, and well known to the general anatomist for the interesting investigations and suggestions made by him on many topics. His work is better arranged and more complete, although somewhat smaller in size, than that of Mandl, recently published. After an introduction on the objects and literature of anatomy and its relations to congenerous sciences, he describes, first, the constituents of the human body; and secondly, the organic systems in general. These he divides into general systems, and special systems, under the former comprising the cellular system; the vascular system, and the nervous system; and under the latter the corneous, the cartilaginous, the osseous, the dental, the fibrous, the muscular, the serous, the cutaneous, and the glandular systems.

The work contains a great amount of information; and, we think, is better worthy of an English version than any of the publications on general anatomy that have recently appeared from the German or French presses. The *Allgemeine Anatomie* of Henle is much more full; but this would be a serious objection to it with the book craft; and it is probable, that if an English translation were published, it would have but a slender sale. The work of M. Bruns is more condensed, and we should like to see it translated on this side of the Atlantic, with the additions, by some competent individual, of what has transpired since it was published in Germany.

---

Charles A. Lee, M. D., of this city, has been appointed Professor of Materia Medica and General Pathology in Geneva Medical College, in place of Professor John Delamater, resigned.—*N. Y. Journal of Medicine, &c.*

